# Seasonal Transition of Active Bacterial and Archaeal Communities in Relation to Water Management in Paddy Soils

**DOI:** 10.1264/jsme2.ME13030

**Published:** 2013-09-05

**Authors:** Hideomi Itoh, Satoshi Ishii, Yutaka Shiratori, Kenshiro Oshima, Shigeto Otsuka, Masahira Hattori, Keishi Senoo

**Affiliations:** 1 Department of Applied Biological Chemistry, Graduate School of Agricultural and Life Sciences, The University of Tokyo, 1–1–1 Yayoi, Bunkyo-ku, Tokyo 113–8657, Japan; 2 Niigata Agricultural Research Institute, 857 Nagakuramati, Nagaoka, Niigata 940–0826, Japan; 3 Department of Computational Biology, Graduate School of Frontier Sciences, The University of Tokyo, 5–1–5 Kashiwanoha, Kashiwa, Chiba 277–8561, Japan

**Keywords:** Paddy soil, Soil microbial diversity, Clone libraries

## Abstract

Paddy soils have an environment in which waterlogging and drainage occur during the rice growing season. Fingerprinting analysis based on soil RNA indicated that active microbial populations changed in response to water management conditions, although the fundamental microbial community was stable as assessed by DNA-based fingerprinting analysis. Comparative clone library analysis based on bacterial and archaeal 16S rRNAs (5,277 and 5,436 clones, respectively) revealed stable and variable members under waterlogged or drained conditions. Clones related to the class *Deltaproteobacteria* and phylum *Euryarchaeota* were most frequently obtained from the samples collected under both waterlogged and drained conditions. Clones related to syntrophic hydrogen-producing bacteria, hydrogenotrophic methanogenic archaea, rice cluster III, V, and IV, and uncultured crenarchaeotal group 1.2 appeared in greater proportion in the samples collected under waterlogged conditions than in those collected under drained conditions, while clones belonging to rice cluster VI related to ammonia-oxidizing archaea (AOA) appeared at higher frequency in the samples collected under drained conditions than in those collected under waterlogged conditions. These results suggested that hydrogenotrophic methanogenesis may become active under waterlogged conditions, whereas ammonia oxidation may progress by rice cluster VI becoming active under drained conditions in the paddy field.

Unlike upland agricultural soils, paddy soils have unique environmental conditions as a result of water management practices (25). In Japan and many other countries, paddy fields are drained completely before rice cultivation and are waterlogged temporally during the rice growing season. As the time of harvest approaches, paddy fields are drained again and maintained in the same condition until the next cultivation season. Such water management practices greatly impact the soil biogeochemical properties of paddy fields. The soil environment is oxic during drained periods, whereas this environment becomes anoxic during waterlogged periods. Various anaerobic biochemical processes occur during waterlogged periods, including nitrate, metal, and SO_4_^2−^ reductions, and methanogenesis (25, 29). Although seasonal transition of the soil geochemical properties of paddy fields have been studied in detail (25), responses of microbial communities to environmental changes in paddy soils are not well understood.

Microbial community shifts have been examined in Italian paddy fields throughout the rice cultivation period (26). It was revealed that the composition of the methanogenic archaeal community was constant during the cultivation period despite seasonal transition of methane production activity. However, these studies were conducted on the basis of the 16S rRNA gene derived from DNA extracted from soils; therefore, the transition of active microbial communities under field conditions has remained unclear. DNA-based gene analysis may detect not only viable but also dead or quiescent cells (19, 36). In contrast, rRNA-based analysis is believed to detect metabolically active populations because growing cells contain more ribosomes and rRNA is degraded immediately after the suspension of metabolic activity (39, 22). Although some microbes are known to maintain the amount of rRNA under starvation conditions (9, 10), previous studies demonstrated that community shifts were more pronounced when identified with an RNA-based approach than with DNA-based analysis (7, 45, 51). Thus, RNA-based analysis may be more suitable to investigate the responses of microbial communities to environmental changes.

RNA-based studies were conducted to investigate bacterial or methanogenic archaeal community shifts in relation to flooded conditions in paddy soil microcosms (38, 35) and Japanese paddy fields (23, 56), indicating some active members among limited groups, bacteria or methanogenic archaea. Flooded paddy fields are well known major environmental sources of methane production, gasses contributing to the greenhouse effect (31). Many previous studies focused on methanogenic archaeal communities in rice paddy soil (12, 33, 26, 57). However, little is known about the transition of whole prokaryotic communities because simultaneous assessment of bacterial and archaeal communities was not performed in any previous field studies based on soil RNA.

Sampling frequency in previous studies may also have limited their assessment of microbial responses to changes in soil geochemical properties (23). Lüdemann *et al.* (32) showed that oxygen was depleted in paddy soil 2.2 mm below the surface after 7 days of incubation. Because the anoxic condition stimulates the anaerobic processes involved in soil geochemical properties, frequent sampling (e.g., weekly) is necessary to assess the responses of active microbial populations to changes in soil geochemical properties.

Consequently, our first objective was to investigate the seasonal transition of both bacterial and archaeal communities in the paddy field in relation to water management during cultivation seasons. On a weekly or biweekly basis, we performed soil sampling and simultaneous assessment of bacterial and archaeal communities by quantitative PCR (qPCR) and fingerprinting analysis using DNA and RNA in the soil samples collected from the paddy fields. The second objective was to identify the active microbial population under waterlogged and drained conditions. Comparative analysis of RNA-based clone libraries was performed to identify the active microbes responding to changes in the soil condition.

## Materials and Methods

### Study site and soil sampling

The paddy field used in this study is located at Niigata Agricultural Research Institute (Nagaoka, Niigata, Japan; 37°26′N, 138°52′E). The soil type is classified as Gley Lowland soil. Rice (*Oryza sativa* L., cv. Koshihikari) has been cultivated in the field since 2003 as a single summer crop from April to September. Water management stages were divided into five categories: before waterlogging (stage BW; until April 29), waterlogging (stage W; April 30–June 18), temporal drainage (stage T; June 19–30), intermittent drainage with cycles of artificial drainage and irrigation (stage I; July 1–August 31), and after complete drainage (stage CD; after September 1). Details of site management in 2009 are described in the supplementary information (Fig. S1).

Soil samples were collected at 20 time points from April to October 2009 (Fig. S1). At each sampling event, 10 soil cores (3 cm in diameter) were collected from the plow layer at a depth of 0 to 10 cm after removing the surface water and mixed well in a plastic bag. Part of the composite sample was immediately frozen in liquid nitrogen, transported with dry ice, and stored at −80°C until used for extraction of nucleic acid. The remaining soil samples were maintained at 4°C until they were used for the analysis of soil characteristics as described below.

### Soil characteristics

Soil moisture and temperature were monitored at a depth of 5 cm in the experimental field every 2 h during the sampling period using an Em5b Analog Data Logger (Decagon Devices, Pullman, WA, USA) equipped with an EC5 soil moisture sensor and ECT temperature sensor (Decagon Devices). Soil Eh was measured at a depth of 5 cm on all sampling dates using three replicate platinum-tipped electrodes and Eh indicator PRN-41 (Fujiwara Scientific, Tokyo, Japan) in the field. N_2_O and CH_4_ flux in the field were measured using the closed chamber method (37). Soil pH and NH_4_-N, NO_3_-N, NO_2_-N, Fe^2+^, Mn^2+^, and SO_4_^2−^ concentrations were measured as described previously (41, 17). Measurement of Fe^2+^ concentration and extraction of Mn^2+^ were performed on the day of sampling to minimize oxidation of the reduced metals. Denitrification and nitrification activity were measured as described in the supporting information.

### Nucleotide preparation

From 1 g (wet weight) of each soil sample, RNA was extracted using the RNA PowerSoil Total RNA Isolation Kit (MoBio Laboratories, Solana Beach, CA, USA) according to the manufacturer’s instructions. DNA was extracted simultaneously through the RNA extraction step using the RNA PowerSoil DNA Elution Accessory Kit (MoBio Laboratories). Crude RNA was purified using the Turbo DNA-free Kit (Applied Biosystems, Foster City, CA, USA) and RNA Clean-Up Kit-5 (Zymo Research, Irvine, CA, USA) to remove DNA and PCR inhibitors as much as possible. First-strand complementary DNA (cDNA) was synthesized by incubating total RNA (100 ng) in 20 μl of reaction mixture composed of Random primer 6 mer (Takara Bio, Otsu, Japan), 5× PrimeScript buffer, SUPERase-In RNase inhibitor (20 units) (Ambion, Austin, TX, USA), and PrimeScript reverse transcriptase (200 units) (Takara Bio) at 42°C for 60 min. The concentration and integrity of the prepared nucleotide solution were determined by spectrophotometry using the NanoDrop ND-1000 spectrophotometer (NanoDrop Technologies, Wilmington, DE, USA), electrophoresis on 1.5% agarose gel stained with ethidium bromide, and qPCR with standard addition. Three separate nucleotide extraction were performed from the original composite soil samples made from 10 soil cores. The reproducibility of replicate nucleotide extractions was confirmed to be similar by denaturing gradient gel electrophoresis (DGGE) (Fig. S2). Three replicates of DNA and cDNA for each soil sample were used for qPCR. Pooled DNA and cDNA from three replicate extractions were used for PCR-DGGE and clone library analyses as described below.

### qPCR

qPCR was performed to amplify bacterial and archaeal 16S rRNA genes using Power SYBR Green PCR Master Mix (Applied Biosystems) and the StepOne system (Applied Biosystems). The reaction mixture comprised 2× SYBR Green PCR Master Mix, 0.2 μM forward and reverse primer pairs ((357F and 520R for bacteria (41); A364a and A934b for archaea (15), 0.5 μg/μl BSA, and DNA or cDNA as a template. The PCR conditions were as follows: initial denaturation at 95°C for 10 min, followed by 40 cycles of 95°C for 30 s, 58°C for 30 s, and 72°C for 30 s (for bacteria) or 40 cycles of 94°C for 30 s, 66.5°C for 30 s, and 72°C for 50 s (for archaea). The amount of archaeal or bacterial 16S rRNA and the 16S rRNA gene copies was calculated on the basis of the standard curve constructed using the dilution series of the plasmid solutions inserted with near full-length 16S rRNA gene sequences from *Methanosarcina barkeri* NBRC 100474 (NR_074253) or *Cupriavidus metallidurans* JCM 21315^T^ (NR_074704), respectively.

### PCR-DGGE

PCR amplification and DGGE were performed as described previously (41, 15). PCR product size was confirmed by electrophoresis on 1.5% agarose gel stained with ethidium bromide, and amplicons were purified using the Wizard SV Gel and PCR CleanUp System (Promega, Madison, WI, USA). Bacterial DGGE bands unique to the sample collected on May 21 were excised from the gel, and DNA was eluted and sequenced as described previously (41).

### Clone library construction and sequencing

For clone library analysis, one DNA sample obtained from the soil sample collected on June 10, 2009, and four cDNA samples obtained from the soil sample collected on April 23, June 10 and 18, and October 1, 2009 were used. Bacterial and archaeal partial 16S rRNAs were amplified under PCR conditions with the optimized PCR cycle numbers, as described in the supporting information. PCR products were verified and purified as described above and were cloned into the pCR TOPO vector using the TOPO TA Cloning Kit (Invitrogen) as described by the manufacturer’s instruction. As a result, 10 clone libraries (bacterial and archaeal 16S rRNA gene sequence libraries from five nucleotide samples) were constructed. From each library, >1000 colonies were randomly picked using the GeneTac G3 Picking System (DIGLAB, Holliston, MA, USA), and their insert sequences were amplified using PCR-1 (5′-GTGCTG CAAGGCGATTAAGTTGG-3′) and PCR-2 (5′-TCCGGCTCG TATGTTGTGTGGA-3′) primers. PCR amplicons were treated with exonuclease I and shrimp alkaline phosphatase (GE Healthcare, Uppsala, Sweden) and sequenced using a BigDye Terminator v3.1 Kit with an automated ABI 3730*xl* capillary sequencer (Applied Biosystems). Bacterial fragments were sequenced from both ends of DNA using primers M13F and M13R (17). High quality sequence data derived from the two primers were assembled, and the consensus sequences were obtained using the Phred/Phrap program (8). Single strand sequencing of archaeal samples was performed using primer M13.

### Phylogenetic analysis

Chimeric sequences from these 16S rRNA clone libraries were detected using the Mallard program with a 99.9% cutoff (1) and removed from the libraries. The resulting sequences were processed for taxonomic assignment at the family and higher levels using RDP Classifier program ver. 2.2 (55) with an 80% confidence threshold. Archaeal or bacterial nucleotide sequences from the five clone libraries were aligned using ClustalW2 (28), and community similarity among the libraries was examined using the Fast UniFrac web interface (13). Rarefaction curves, Chao1 and ACE richness indices, and Shannon and Simpson’s diversity indices were calculated using Mothur program ver. 1.14.0 (47). Sequences from each library were also clustered into OTUs with 3% differences using the Mothur program. OTUs specific to each library and shared OTUs between two libraries were identified using the template match method with a similarity index of <0.10 as a cutoff value (16, 17) using R software ver. 2.10.1. (http://www.r-project.org/). Phylogenetic tree was constructed using the neighbor-joining method with bootstrap test (1,000 replicates) using MEGA ver. 4.0.2 (53).

### Statistical analysis

Analysis of variance (ANOVA) was performed using R software ver. 2.10.1 to analyze differences in soil characteristics and qPCR data among samples. R software was also used to perform the principal component analysis (PCA) of the DGGE profiles digitized using CS Analyzer 3 (ATTO, Tokyo, Japan) (16) and Fisher’s exact test to analyze taxonomic distributions among the clone libraries.

### Nucleotide sequence accession number

The nucleotide sequences reported in this paper were deposited in the DDBJ/Genbank/EBI databases under accession numbers AB661339–AB661347 (DGGE bands) and AB650607–AB661319 (sequences obtained from the clone libraries).

## Results

### Seasonal transition of soil biogeochemical properties

**Fig. 1:**
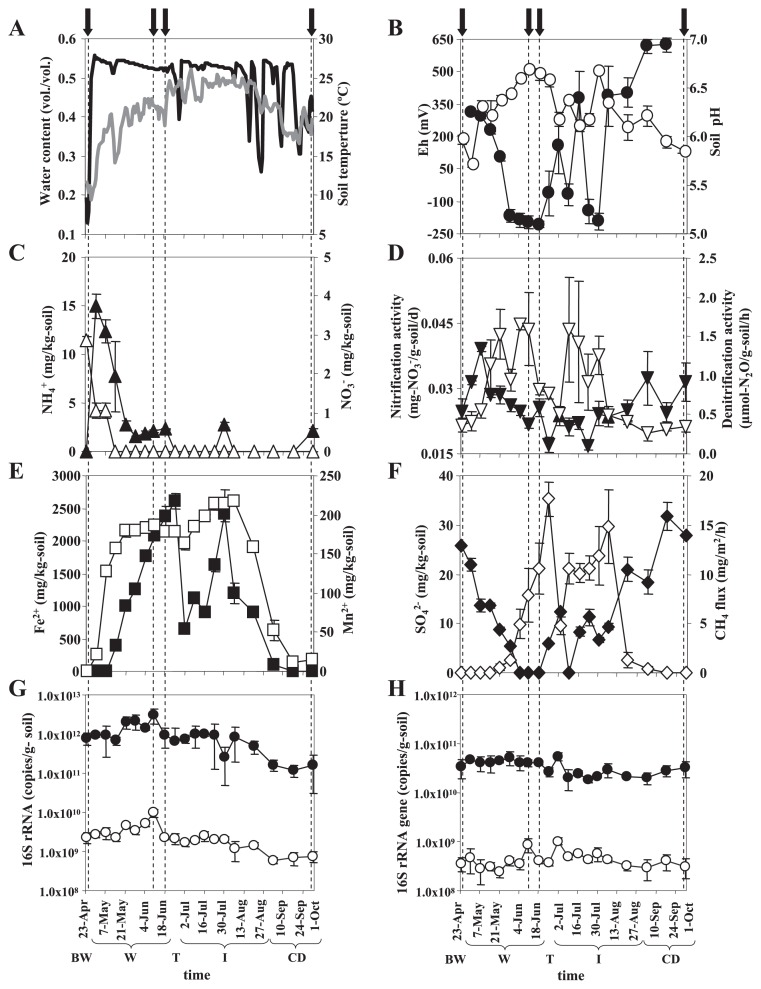
Seasonal transition of soil biogeochemical properties in paddy soils for the sampling period. (A) Daily average soil water content (black line) and soil temperature (gray line) at 5-cm depth in the experimental paddy field; (B) soil redox potential (Eh) (closed circles) and pH (open circles) at 5-cm depth; (C) concentrations of NH_4_^+^ (closed triangles) and NO_3_^−^ (open triangles); (D) nitrification activity (closed reverse triangles) and denitrification activity (open reverse triangles); (E) concentrations of Fe^2+^ (closed squares) and Mn^2+^ (open squares); (F) concentrations of SO_4_^2−^ (closed diamonds) and CH_4_ flux from the experimental field (open diamonds); (G) amount of bacterial (smaller closed circles) and archaeal (smaller open circles) 16S rRNAs measured by RNA-based qPCR; and (H) copy number of bacterial (smaller closed circles) and archaeal (smaller open circles) 16S rRNA genes measured by DNA-based qPCR. Mean ±SD is shown (n=3). Samples as indicated with arrows and dotted lines were used for clone library analysis. Letters shown at the bottom indicate the water management stages: before waterlogging (stage BW; until 29 April), waterlogging (stage W; April 30–June 18), temporal drainage (stage T; June 19–30), intermittent drainage with cycles of artificial drainage and irrigation (stage I; July 1–August 31), and after complete drainage (stage CD; after September 1) (see Fig. S1).

During the sampling period, we measured 14 soil biogeochemical properties. Volumetric water content in paddy soil increased after waterlogging, reached a plateau (>50%) during waterlogged periods (stage W; April 30–June 18), and decreased with water drainage (Fig. 1A). Soil Eh decreased gradually after waterlogging and was maintained at approximately −230 mV at a later stage W (June 4–18; Fig. 1B). Soil Eh increased in response to water drainage and was maintained at >+350 mV after complete drainage (stage CD; after September 1) (Fig. 1B). NH_4_^+^ concentration increased after fertilizer application on April 23 and decreased thereafter (Fig. 1C). NO_3_^−^ concentration was low before waterlogging (stage BW; until April 29) and became <1 mg N kg^−1^ soil by 2 weeks after waterlogging (on April 30; Fig. 1C). Nitrite was not detected (<1 mg NO_2_^−^-N kg^−1^ soil) during the sampling period (data not shown). Potential nitrification activity increased in the initial stage W (April 30–May 21) and in stage CD (Fig. 1D). Potential denitrifying activity changed in accordance with soil Eh (Fig. 1D). Similarly, concentrations of Mn^2+^ and Fe^2+^ and CH_4_ flux increased and SO_4_^2−^ concentration decreased in accordance with the changes in soil Eh (Figs. 1E and 1F). N_2_O flux from the paddy field was low (<0.1 μg N_2_O-N m^−2^ h^−1^) throughout the sampling period (data not shown). Fig. 1G shows the results of qPCR analysis performed using RNA extracted from soils. The amounts of bacterial 16S rRNA were similar among stage BW, W, T, and I with no significant different (*P*>0.05). However, they decreased from stage W to stage CD, and the amounts during stages W and CD were significantly different (*P*<0.05). A similar tendency was also observed during analysis using archaeal 16S rRNA (Fig. 1G). In contrast, qPCR analysis using DNA extracted from soils showed that the amount of bacterial and archaeal 16S rRNA genes did not change throughout the sampling period (Fig. 1H).

### Seasonal transition of the structure of bacterial and archaeal communities throughout the cultivation period

**Fig. 2:**
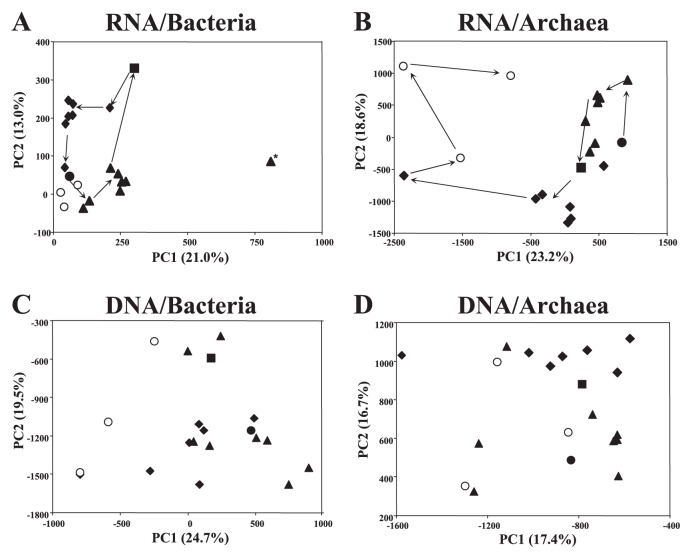
Transition of bacterial and archaeal community structures as assessed by principal component analysis (PCA) based on the denaturing gradient gel electrophoresis (DGGE) profiles. PCA plots based on (A) RNA-based DGGE targeting bacterial 16S rRNA; (B) RNA-based DGGE targeting archaeal 16S rRNA; (C) DNA-based DGGE targeting bacterial 16S rRNA genes; and (D) DNA-based DGGE targeting archaeal 16S rRNA genes are shown. Legend: closed circle, stage BW; closed triangles, stage W; closed square, stage T; closed diamonds, stage I; open circles, stage CD. Arrows indicate the temporal transition sequences. Values in parentheses show the percentage of community variation explained by each component. The symbol with asterisk in panel A indicates the profiles derived from the sample collected on May 21 (see Supplementary information).

Based on the DGGE profiles targeting bacterial 16S rRNA, the bacterial community structure appeared to be similar among samples (Fig. S3A); however, soil samples in each water management stage were grouped together while examining the band intensities (Fig. 2A). The DGGE profiles of samples in stages BW and CD, in which the soils were both under drained conditions, were close together on the PCA plot (Fig. 2A). The DGGE profile of the sample collected on May 21 was distinct from that of the other samples (Figs. 2A and S3A). Bands unique to this profile were excised and sequenced. Phylogenetic analysis of the sequences from seven major bands showed that the sequences of band A belonged to the phylum *Betaproteobacteria* (Table S1) and those of bands B to G were related to the phylum *Cyanobacteria*, not chloroplasts (Table S1 and Fig. S4). Similar to the DGGE profiles targeting bacterial 16S rRNA, DGGE profiles targeting archaeal 16S rRNA were similar across samples (Fig. S3B). However, PCA analysis of band intensities showed that the archaeal DGGE profiles changed with time (Fig. 2B). In addition to the analysis based on RNA, we performed DGGE analysis on the basis of DNA extracted from soils. In contrast to the results of RNA-based analysis, bacterial and archaeal community structures were stable over time (Figs. S3C and S3D), and no apparent groupings of samples were observed on the PCA plots (Figs. 2C and 2D).

### Comparative clone library analysis

**Fig. 3:**
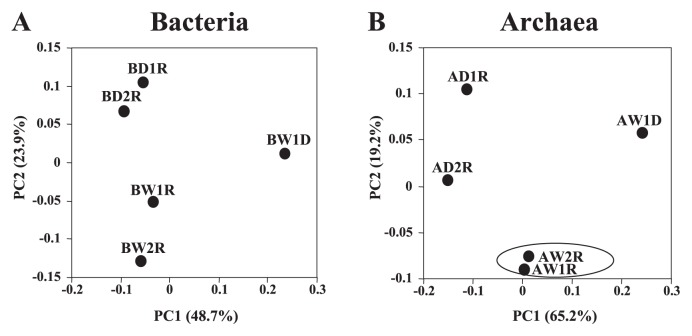
Principal coordinate analysis (PCoA) plots based on (A) bacterial and (B) archaeal clone libraries. PCoA plots were generated using Fast UniFrac analysis based on the weighted algorithm with normalization. Values in parentheses show the percentage of community variation explained by each coordinate. Samples in circles have significant similarity (*P*>0.05). The first letter of library ID (A or B) represents the target community: B, bacteria; A, archaea. The final letter of library ID (D or R) represents the nucleic acid used for analysis: D, DNA; R, RNA. Libraries with ID containing “D1,” “W1,” “W2,” and “D2” were derived from soil samples collected on Apr. 23, June 10, June 18, and October 1, respectively. So, D1 and D2 libraries were derived from drained conditions, while W1 and W2 libraries were derived from waterlogged conditions.

qPCR and DGGE analyses indicated that active populations in bacterial and archaeal communities were different between stages W and CD (Fig. 2). On the basis of these data, we selected four RNA samples (D1R at stage BW, W1R and W2R at stage W, and D2R at stage CD) derived from soil samples collected on April 23, June 10, June 18, and October 1, respectively, for further analysis. In addition, one DNA sample, W1D in stage W, was used for further analysis. We performed comparative clone library analysis to identify active populations in bacterial and archaeal communities and their seasonal dynamics in detail. The Mallard program (1) detected 2.3 to 5.7% and 0.6 to 3.7% of the sequences as chimeras from bacterial and archaeal clone libraries, respectively. After removal of these chimeric sequences, we obtained 5,277 and 5,436 sequences from bacterial and archaeal clone libraries, respectively (Table 1), and used them for subsequent analysis. The diversity of communities derived from each library was analyzed using the Mothur program (47). At 3% difference, 692 to 731 and 132 to 191 operational taxonomic units (OTUs) were detected from bacterial and archaeal libraries, respectively (Table 1). The Chao1 and ACE values as well as Shannon and Simpson diversity indices were higher in bacterial libraries than in archaeal libraries (Table 1). Library coverage (Cx) values ranged from 43 to 54% and 88 to 93% in bacterial and archaeal libraries, respectively. Similarities among libraries analyzed by weighted Fast UniFrac (13) suggested that libraries constructed from DNA were distinguished from those constructed from RNA (Figs. 3A and 3B), regardless of the target molecule (*i.e.*, bacterial or archaeal 16S rRNA). AW1R and AW2R libraries were very close to each other (Fig. 3B). The difference between AW1R and AW2R was not significant based on Fast UniFrac *P* test with 500 permutations (*P*=0.2), while differences among the other bacterial and archaeal libraries were significant (*P*<0.05).

**Fig. 4:**
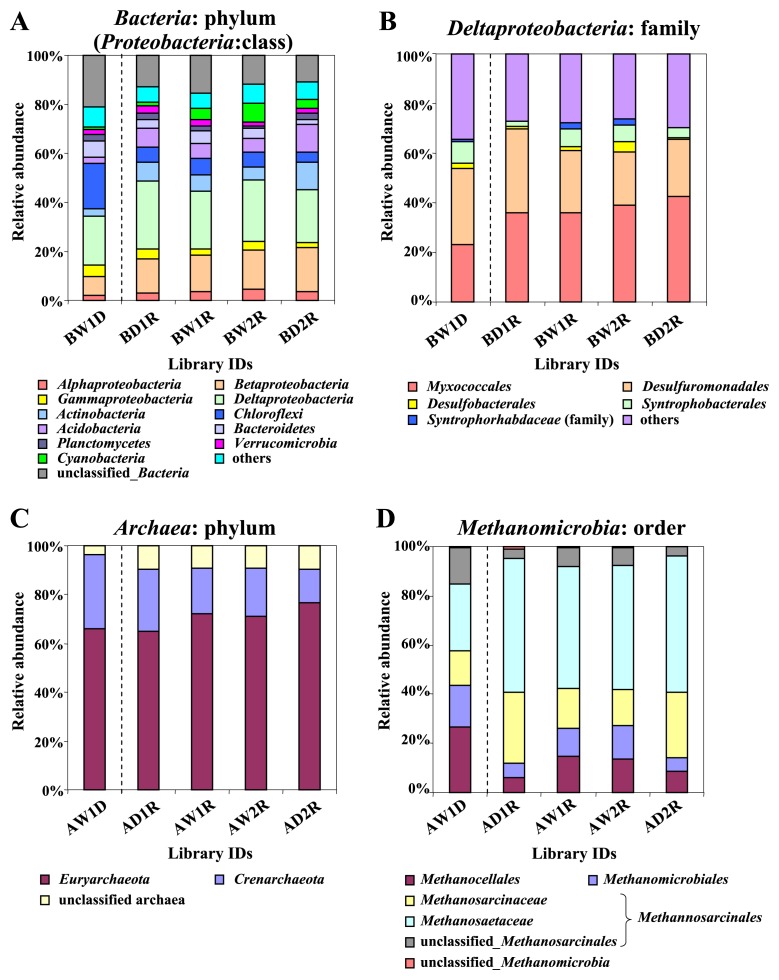
Relative distributions of the clones at different taxonomic resolutions. Resolutions at (A) bacterial phylum and proteobacterial class; (B) deltaproteobacterial order; (C) archaeal phylum; and (D) *Methanomicrobia* order and *Methanosarcinales* family are shown. The order name for the family *Syntrophorhabdaceae* is unidentified (43). Sequences were classified using the RDP Classifier with a threshold level of 80%.

Fig. 4A shows the phylum- or class-level distribution of clones obtained from the libraries targeting bacterial 16S rRNA. *Deltaproteobacteria* dominated among bacteria in all four samples derived from RNA. The relative abundances of the phylum *Cyanobacteria* and *Bacteroidetes* seemed to increase in the BW1R and BW2R libraries constructed using soil samples at stage W than in the BD1R and BD2R libraries constructed using soil samples at stage BW and CD, although with no significant difference (*P*>0.05) based on Fisher’s exact test. Within the deltaproteobacterial clones, clones belonging to the order *Myxococcales* were dominant, ranging from 35.6 to 42.5% (Fig. 4B); however, their proportions did not change with library type. In contrast, proportions of the orders *Desulfobacterales*, *Syntrophobacterales*, and the family *Syntrophorhabdaceae* varied with library type and were greater (*P*<0.05) in the libraries constructed using soil samples in stage W than in those constructed using other samples (Fig. 4B). Fig. 4C shows the phylum-level distribution of clones obtained from libraries targeting archaeal 16S rRNA. *Euryarchaeota* dominated throughout the sampling period with their proportions ranging from 65.5 to 76.7% (Fig. 4C). Most (90.9 to 95.1%) of the euryarchaeal sequences belonged to the class *Methanomicrobia*, which includes the orders *Methanocellales*, *Methanomicrobiales*, and *Methanosarcinales*. The relative abundances of *Methanocellales* and *Methanomicrobiales* were 1.9 to 2.3 times higher (*P*<0.05) in the libraries constructed using soils under waterlogged conditions (AW1R and AW2R) than in those constructed using soils under drained conditions (AD1R and AD2R) (Fig. 4D), respectively. Because we constructed clone libraries using DNA and RNA extracted from the same soil samples, we could compare the taxonomic distributions of clones between the two libraries (W1D and W1R). Fig. 4 shows that the taxonomic distributions were distinct from each other, similar to the results of Fast UniFrac analysis described above. More detailed taxonomic compositions are shown in Tables S3 and S4.

### Identification of active populations in bacterial and archaeal communities in paddy soils under waterlogged or drained conditions

**Fig. 5:**
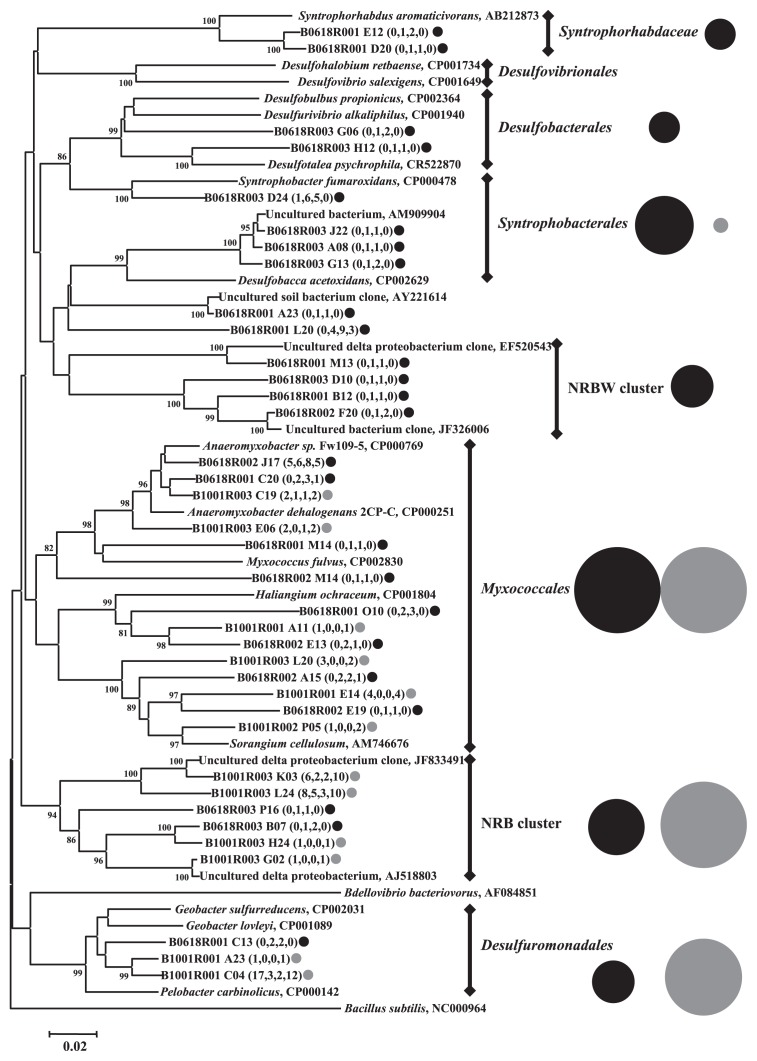
Phylogenetic relatedness of the deltaproteobacterial 16S rRNA recovered from representative sequences from specific OTUs. Sequences IDs obtained in this study are indicated by colored smaller circles, black (OTUwB) or gray (OTUdB). Numbers in parentheses are the numbers of clones in each OTUs that originated from the four libraries (BD1R, BW1R, BW2R, and BD2R). The size of right larger circle indicates relative abundance of the total number of clones belonging to each cluster and the circle colors indicate association to either OTUwB or OTUdB as well as sequence ID. NRBW and NRB clusters indicate the unidentified clusters with less similarity to known sequences despite their frequent detection in our experimental paddy soil. Bootstrap values (>70%) with 1,000 replicates are shown next to the branches. The 16S rRNA sequence of *Bacillus subtilis* (NC_00964) was used as an outgroup.

The results of DGGE and clone library analyses indicated that active populations in bacterial and archaeal communities in paddy soils may have changed in response to water management conditions. To identify the microbes responsive to waterlogged or drained soil conditions, we performed OTU-based analysis. Using the Mothur program and the template match method (16, 17), we identified OTUs specific to the libraries (W1R and W2R) that were constructed using soils under waterlogged conditions (OTUw) and those specific to the libraries (D1R and D2R) that were constructed using soils under drained conditions (OTUd). In the bacterial clone libraries, 95 and 78 OTUs were identified as being OTUw and OTUd (OTUwB and OTUdB), respectively. The OTUwB was dominated by *Deltaproteobacteria* (26.3%), *Betaproteobacteria* (14.7%), and *Cyanobacteria* (8.4%), while the OTUdB was dominated by *Deltaproteobacteria* (15.4%), *Actinobacteria* (15.4%), and *Betaproteobacteria* (12.8%) (Table S2). Among *Deltaproteobacteria*, *Desulfobacterales*, *Syntrophobacterales*, *Syntrophorhabdaceae*, and the NRBW cluster (unidentified cluster detected frequently in our experimental paddy soil) appeared only in OTUwB (Fig. 5).

**Fig. 6:**
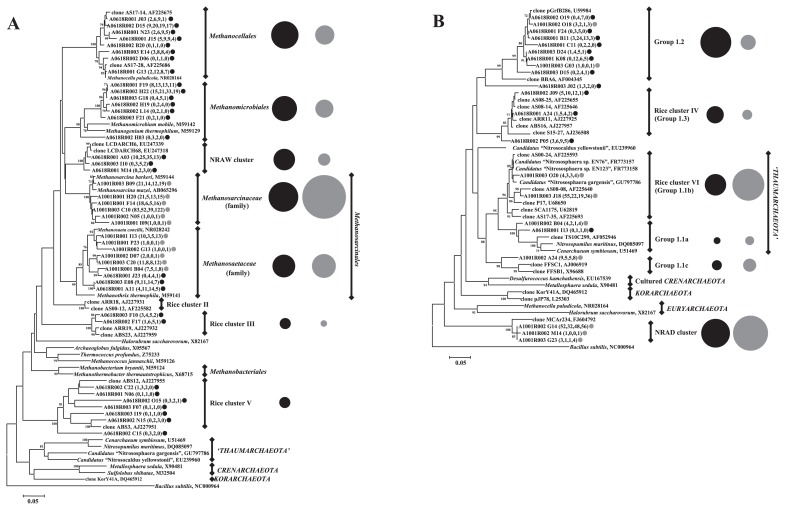
A. Phylogenetic relatedness of the euryarchaeotal 16S rRNA recovered from representative sequences from specific OTUs. Sequences ID obtained in this study are colored black (OTUwA) or gray (OTUdA). Numbers in parentheses are the numbers of clones in each OTUs originating from the four libraries (AD1R, AW1R, AW2R, and AD2R). Circle size indicates the relative abundance of the total number of clones belonging to each cluster, and the circle colors indicate the deviation of OTUwA or OTUdA as well as sequence ID. NRAW cluster indicates the unidentified cluster with less similarity to known sequences despite frequent detection under waterlogged conditions in our experimental paddy soil. Bootstrap values (>70%) with 1,000 replicates are shown next to the branches. The 16S rRNA sequence of *Bacillus subtilis* (NC_00964) was used as an outgroup. B. Phylogenetic relatedness of crenarchaeal and unclassified archaeal 16S rRNA recovered from representative sequences from specific OTUs. NRAD cluster indicates the unidentified cluster with less similarity to known sequences despite frequent detection under drained conditions in our experimental paddy soil.

Similar analysis was performed with OTUs obtained from the archaeal clone libraries. Forty-two and 21 OTUs were identified as being OTUw and OTUd (OTUwA and OTUdA), respectively. Among these, 67.2% and 57.1% of OTUwA and OTUdA, respectively, were assigned as *Euryarchaeota* by the Ribosomal Database Project (RDP) Classifier ver. 2.2 (55). Fig. 6A shows the phylogenetic tree constructed on the basis of the representative sequences from OTUwA and OTUdA belonging to *Euryarchaeota*. These OTUwA were distributed across *Methanocellales*, rice cluster III and V, the families *Methanomicrobiaceae*, *Methanosaetaceae*, and the NRAW cluster (unidentified cluster detected frequently in our experimental paddy soil). In contrast, OTUdA appeared frequently in *Methanosarcinaceae* and *Methanosaetaceae* of *Methanosarcinales*. In addition to *Euryarchaeota*, 32.8% and 42.9% of OTUwA and OTUdA, respectively, were assigned as the phylum *Crenarchaeota* or unclassified archaea by the RDP Classifier ver. 2.2. The phylogenetic relationship among the representative sequences from OTUwA and OTUdA belonging to *Crenarchaeota* or unclassified archaea was also examined (Fig. 6B). These OTUwA were related to uncultured *Crenarchaeaota* Group 1.2, Group 1.1a, and rice cluster IV, whereas OTUd were related to Group 1.2, rice cluster VI, Group1.1a, Group1.1c, and the NRAD cluster (unidentified cluster detected frequently in our experimental paddy soil). The majority (65.0%) of the sequences from OTUwA were related to Group 1.2, whereas only 3.2% of the sequences from OTUdA were related to this cluster. In contrast, many (39.8%) of the sequences from OTUdA were related to rice cluster VI.

## Discussion

We showed the seasonal transition of soil biogeochemical properties in our experimental paddy field throughout the cultivation period (Fig. 1). During stage W, soil Eh decreased gradually, and anaerobic processes such as denitrification, metal and SO_4_^2−^ reductions, and methanogenesis progressed sequentially, as also observed in previous studies (25, 59, 33). In contrast, these anaerobic processes were depressed under drained conditions. Finally, soil biogeochemical properties of the samples in stage CD were close to those in stage BW. The observed seasonal transition of soil characteristics confirmed that the soil environment became aerobic in stages BW and CD and anoxic at stage W, especially in the later stage (June 4–18) (Fig. 1). Based on the seasonal transition of the amount of 16S rRNA, both bacteria and archaea were suggested to be more abundant in stage W than stage CD (Fig. 1G). Similar to our results, Conrad and Klose also showed that the copy numbers of bacterial and archaeal 16S rRNA genes increased in paddy soil microcosms amended with rice straw after waterlogging (6). Our experimental field received rice straw in October 2008; however, most remained undegraded in soil in spring before cultivation in 2009 because of the low temperature (<6°C, daily average air temperature) in winter. Microbes could use the remaining rice straw to support their growth in stage W under warm condition (24). Under the drained condition in stage CD, the decrease of prokaryotic 16S rRNA might be affected by a longer drought period and lower temperature (Fig. 1G).

The results of RNA-based DGGE analysis and clone library analysis suggested that active populations in bacterial and archaeal communities responded to water management conditions, although overall community structures were stable over time as identified by DNA-based DGGE analysis (Figs. 2 and 3). Previous field studies based on DNA or phospholipids fatty acids directly extracted from soils did not identify the effects of water management on the potential structures of bacteria and methanogenic archaea in paddy soils (23, 40, 56). These results suggested that RNA-based analysis is more sensitive for detecting active populations in the paddy field soil responsive to specific stimuli (*e.g.*, water management conditions). Indeed, some studies based on the soil microcosm could detect the transitions of structure of microbial communities in paddy soil by the DNA-based method. However, they constructed the soil microcosm by adding some carbon or nitrogen sources or waterlogged with air-dried soil to trace the transition of structure of microbial communities (17, 33). Compared with the natural conditions, these stimulations might be more extreme, so changes to the structure of microbial communities were suggested to be detectable even through the DNA-based approach. Moreover, the PCA plots of bacterial DGGE profiles based on RNA at stage BW and CD were closer together than those of archaeal DGGE profiles, suggesting that the structure of active bacterial communities may recover more quickly from waterlogging stimuli than those of archaeal communities in the paddy field. Although their growth rate or resistance to oxygen may affect the difference, further studies with replicates are needed.

Comparative clone library analysis showed that clones related to *Deltaproteobacteria* were most abundant in all samples (Fig. 4A), in contrast to the bacterial community structure in other soil environments (18, 49). Most *Deltaproteobacteria* are known to be strict anaerobes, except some groups related to *Myxococcales* (27). *Deltaproteobacteria* were suggested to dominate in paddy soil due to the temporal anoxic condition during the waterlogged period. *Methanosarcinales* were also most abundant in all samples (Fig. 4D), which are strict anaerobes producing methane (5). Some members related to *Methanosarcinales* appeared at high frequency despite the oxic condition under drained conditions in stage BW and CD, when methane flux was undetectable (Figs. 1F and 6A). Other studies based on 16S rRNA genes and their transcripts also reported that the structure of methanogenic archaea was maintained during the cultivation season and became stable even in the drained period when methane flux was not detected (56, 35). Liu showed that methanogens were fragile with oxygen in the medium but survived even under oxic conditions if given the soil particles (30). Anaerobic *Deltaproteobacteria* and methanogens were suggested to survive (may be resting) even during the drained seasons in the paddy soil, maintaining a certain amount of rRNA, as reported in some bacteria (9, 10).

Within *Deltaproteobacteria*, clones related to *Desulfobacterales*, *Syntrophobacterales*, and *Syntrophorhabdaceae* increased under waterlogged conditions (Figs. 4B and 5). Considering the increase of bacterial population size under waterlogged conditions indicated by qPCR analysis, the amount of these groups was suggested to increase under waterlogged conditions. These bacteria can use SO_4_^2−^ or protons as electron acceptors (42, 43). Their clones were frequently detected in other paddy soils (58, 34), suggesting that these *Deltaproteobacteria* may play an important role in SO_4_^2−^ reduction and hydrogen production under anoxic conditions in paddy fields. The anoxic environment caused by waterlogging may also develop an anaerobic archaeal community in paddy soils. Although *Methanosarcinales* clones were the most dominant in all soil samples, the proportions of clones related to *Methanomicrobiales* and *Methanocellales* increased in soils under anoxic conditions (Figs. 4D and 6A). Because *Methanomicrobiales* and *Methanocellales* archaea are known to be hydrogenotrophic methanogens (11, 46), they most probably produce methane from CO_2_ and H_2_ in paddy soils. Previous *in vitro* experiments indicated that the occurrence of hydrogenotrophic methanogenesis was supported by the presence of *Syntrophobacterales* and *Syntrophorhabdaceae*, which are H_2_ producers and require H_2_-utilizing organisms, such as methanogens and SO_4_^2−^ reducers, as syntrophic partners (20, 43). On June 10 and 18, SO_4_^2−^ concentration became undetectable and methane flux increased greatly (Fig. 1F); therefore, H_2_ produced by *Syntrophorhabdaceae* and *Syntrophobacterales* was most probably consumed by methanogenic archaea rather than SO_4_^2−^ -reducing bacteria. A recent microcosm study also showed the increase of the relative abundance of hydrogenotrophic methanogens related to the family *Methanomicrobiaceae* (order *Methanomicrobiales*) and *Methanocellaceae* (order *Methanocellales*) in paddy soil after waterlogging, based on terminal restriction fragment length polymorphism analyses of *mcrA* transcripts encoding the alpha subunit of methyl coenzyme M reductase involved in methane production reaction (35). Combined with these results, *Methanomicrobiales* and *Methanocellales* are suggested to increase their cell numbers through methanogenesis more rapidly than other methanogenic archaea. Ma et al. reported that the *Methanocellales* organism has a unique set of genes encoding antioxidant enzymes on its genome (35). It is possible that *Methanocellales* have some tolerance to oxygen, although the other methanogens do not, and could grow advantageously under waterlogged conditions after the drained period.

In addition to hydrogenotrophic methanogenic archaea, rice clusters III, IV, and V were revealed to become more active under waterlogged conditions. These clusters were detected frequently in Italian and Asian paddy soils in previous studies (33, 21, 44), although there is no information about their seasonal transition. Although the ecological function of these rice clusters remains unclear, these rice clusters may prefer anoxic conditions and have anaerobic respiration systems to survive under anoxic conditions in paddy soils.

In contrast to soils under waterlogged conditions, clones related to rice cluster VI (equivalent to Group 1.1b) appeared at high frequency under drained conditions (Fig. 6B). Considering the similar amount of archaea in stage BW and W (Fig. 1G), rice cluster VI was suggested to become active under oxic conditions, before waterlogging at least. Representative sequences belonging to rice cluster VI were closely related to *Candidatus* Nitrososphaera sp., an ammonia-oxidizing archaea (AOA) (14, 53). Recently, Group 1.1a and 1.1b containing AOA isolates were proposed to represent a new archaeal cluster, the phylum *Thaumarchaeota* (2, 50). Similar to our study, clones of ammonia monooxygenase gene (*amoA*) for AOAs were also obtained from both rhizosphere and bulk soils in paddy fields (3). In addition, clones related to rice cluster VI were frequently retrieved from paddy soils before waterlogged incubation in a microcosm study (4). Rice cluster VI may become active under drained conditions and play an important role in ammonia oxidation in paddy soil.

This study showed the seasonal transition of active populations in bacterial and archaeal communities in response to marked changes in soil biogeochemical properties in paddy fields. Simultaneous assessment of bacterial and archaeal communities indicated that hydrogenotrophic methanogenesis communities and rice clusters III, IV, and V became active under waterlogged conditions, but they were depressed under drained conditions in the paddy field. This study also showed that rice cluster VI related to AOA tended to appear at high frequency under drained conditions, whereas both DNA- and RNA-based analysis of 16S rRNA indicated that the fundamental microbial community was stable despite the marked changes of soil geochemical properties. Although RNA molecules are thought to be an indicator of active microbes, some researchers have reported organisms maintaining a constant amount of rRNA under starvation conditions, as described above (8, 9). mRNA molecules could be a stricter indicator of active microbes since the half-life of mRNA is extremely short (48). Further studies with more replicates and frequent sampling are needed, and metatran-scriptomic approaches covering not only rRNA sequences but also mRNA sequences may improve our understanding of the seasonal transition of active microbes in paddy fields (54).

## Supplementary Material



## Figures and Tables

**Table 1:** Diversity properties of bacterial and archaeal clone libraries

Target	Library ID	No. of sequences^a^	No. of OTUs^b^	Cx^c^	Estimated OTUs richness^d^		Diversity indices	
	
					Chao1	Ace	Shannon^e^	1/Simpson
Bacteria	BW1D	999	708	0.43	2623 (2169, 3216)	5719 (5084, 6447)	6.33±0.06	571
	BD1R	1069	693	0.52	1843 (1580, 2183)	3382 (3034, 3782)	6.30±0.06	569
	BW1R	1058	731	0.48	1990 (1712, 2349)	3539 (3167, 3967)	6.42±0.05	859
	BW2R	1059	725	0.48	2115 (1802, 2519)	3914 (3493, 4398)	6.39±0.06	738
	BD2R	1092	692	0.54	1802 (1547, 2134)	2960 (2660, 3305)	6.31±0.06	622
Archaea	AW1D	1085	134	0.93	341 (236, 553)	528 (440, 643)	3.92±0.07	32
	AD1R	1063	191	0.88	857 (544, 1444)	1539 (1328, 1790)	4.04±0.09	28
	AW1R	1075	189	0.89	581 (408, 892)	1078 (923, 1267)	4.24±0.08	42
	AW2R	1107	176	0.92	463 (332, 706)	647 (551, 770)	4.23±0.07	43
	AD2R	1106	132	0.93	572 (332, 1101)	731 (613, 879)	3.62±0.09	19

a Number of sequences after removal of chimeric sequences by Mallard.

b Calculated with Mothur at the 3% distance level.

c Calculated from the equation, Cx=1-(n/N), where ‘n’ is the number of OTUs composed of only one sequence (singleton) and N is the total number of sequences.

d Numbers in parentheses are 95% confidence intervals.

e Mean ±95% confidence intervals.
