# Supplementary material for: Seasonal Transition of Active Bacterial and Archaeal Communities in Relation to Water Management in Paddy Soils
Source: Microbes Environ. 2013 Sep 5;28(3):370–80. doi: 10.1264/jsme2.ME13030 (PMC4070958; doi:10.1264/jsme2.ME13030)
Supplement: Supplementary file 1 [file 28_370_s1.pdf]

Supplementary Information

Supplementary experimental procedures

Site management

Our experimental field received the incorporation of rice straw in October 2008. Chemical fertilizer (ammonium sulfate 30 kg/ha) was applied to the field on April 23, 2009. Right after the fertilizer application, the plow layer (*ca*. 17 cm) was mixed by rotary tiller. The field was tilled again and submerged under water on April 30. Rice seedlings were transplanted on May 12, 2009. The first herbicide containing 15% (w/w) bromobutide, 7.5% (w/w) fentrazamide, and 1.3% (w/w) bensulfuron-methyl was applied on May 20 and the second one containing 1.8% (w/w) cyhalofop-butyl on May 25. During rice cultivation, temporal drainage (June 19–30) and intermittent irrigation (July 1–August 31) with cycles of artificial drainage and irrigation were performed in summer in order to suppress CH_4 emissions (1). Additional fertilizer (ammonium chloride 10 kg/ha) applications were performed on July 17 and 24. Water was completely drained on September 1, and rice plants were harvested on September 22, 2009.

Measurement of denitrification and nitrification activity.

Denitrification activity of the soil samples was measured with the acetylene block technique (2). The reaction mixture comprised 1 g (dry weight) fresh soil, 2 ml sterilized water, and 50 µmol $NaNO_3$ in a 10-ml glass vial. The vial was sealed with a butyl rubber cap and the headspace gas was substituted to Ar-C_2H_2 (90:10 (v/v)) gas. After incubation at 20°C for 24 h, the amount of N_2O produced in the headspace was measured by gas chromatography as described previously (Saito *et al*., 2008). Nitrification activity was assayed by measuring the amount of nitrate produced after incubating 1.5 g (dry soil basis) fresh soil at 20°C with 15 ml sterilized water and 30 µmol $(NH_4)_2SO_4$ in a 100-ml glass flask for 2 weeks under aerobic conditions.

Optimization of number of PCR cycles for clone libraries.

In order to minimize PCR bias in the clone library analysis, number of PCR cycles were optimized by qPCR with quenching fluorescence-labeled primer (Q-primer) (3). Primers Q-10F (5'-CAGTTTGATYMTGGCTCAG-3') and Q-934R (5'-CTGCTCCCCCGCCAATTCCT-3'; 4) were labeled with fluorescence molecule at their 5'-end site (J-Bio21, Tsukuba, Japan). Primers Q-10F and 907R (5), and A364a (4) and Q-934R were used for the amplification of bacterial and archaeal 16S rRNA genes, respectively. The 20 µl reaction mixture comprised 1x Ex Taq Buffer, 2.5 mM dNTP,

0.1 µM Q-primer, 0.1 µM non-labeled primer, 0.5 µg/µl BSA, and Ex Taq HS polymerase (Takara Bio, Otsu, Japan). The qPCR conditions were as follows: initial denaturation at 95°C for 5 min, followed by 45 cycles of 95°C for 30 s, primer annealing (54°C and 64 °C for bacteria and archaea, respectively) for 45 s, and 72°C for 60 s for bacteria (45 s for archaea). Based on the results of the qPCR with Q-primers, optimal PCR cycle numbers were identified as being 16 and 19 for bacteria and archaea, respectively. Bacterial and archaeal partial 16S rRNA were amplified under the same PCR conditions with the optimal PCR cycle numbers.

References

1. Uoki, Y., and S. Noda. 2001. Suppression of methane emission from Strong-Gley soils paddy field by water managements. Japanease Journal Soil Science and Plant Nutrition 72:449-452 (In Japanese).
2. Tiedje, J. M. 1994. Denitrifiers, p. 245-267. *In* R.W. Weaver, J.S. Angle, P.J. Bottomley (ed.), Methods of soil analysis part 2. Microbiological and biochemical properties, Soil Science Society of America Inc., Wisconsin.
3. Nishizawa, T., M. Komatsuzaki, N. Kaneko, and H. Ohta. 2008. Archaeal diversity of upland rice field soils assessed by the terminal restriction fragment length polymorphism method combined with real time quantitative-PCR and a clone library analysis. Microbes Environ. 23:237-243.
4. Kemnitz, D., S. Kolb, and R. Conrad. 2007. High abundance of *Crenarchaeota* in a temperate acidic forest soil. FEMS Microbiol. Ecol. 60:442-448.
5. Engelbrektson, A., V. Kunin, K.C. Wrighton, N. Zvenigorodsky, F. Chen, H. Ochman, and P. Hugenholtz. 2010. Experimental factors affecting PCR-based estimates of microbial species richness and evenness. ISME J. 4:642-647.

Table S1. Close relatives of sequences of the DGGE bands excised from polyacrylamide gel showed in Figure S2.

		Closest reference sequences			
DGGE band	Microorganisms	Phylogenetic affiliations	Accession number	Identity (%)	Alignment
A	*Methylibium* sp.	*Betaproteobacteria/Burkholderiales*	AB609313	100	196/196
B1	*Anabaena* sp.	*Cyanobacteria/Nostocales*	FJ948087	98.8	171/173
B2	*Cylindrospermum* sp.	*Cyanobacteria/Nostocales*	GU055195	98.8	171/173
C	*Anabaena* sp.	*Cyanobacteria/Nostocales*	FN691915	98.9	174/176
D	*Anabaena* sp.	*Cyanobacteria/Nostocales*	HE975017	100	164/164
E	*Cylindrospermopsis raciborskii*	*Cyanobacteria/Nostocales*	JQ707296	100	174/174
F1	*Anabaena cylindrica*	*Cyanobacteria/Nostocales*	HE975014	100	174/174
F2	*Anabaena cylindrica*	*Cyanobacteria/Nostocales*	HE975014	99.4	173/174
G	*Nostoc* sp.	*Cyanobacteria/Nostocales*	JF810617	100	174/174

Table S2. Taxonomic composition of the specific OTUs in the bacterial clone libraries.

**Phylogenic group**	**Number of OTUs (Number of clones)[a]**			
	**OTUwB[c]**		**OTUdB[d]**	
Proteobacteria	**47**	**(10,83,92,12)**	**30**	**(96, 17, 20, 94)**
Alphaproteobacteria	6	(0,7,8,0)	4	(5,0,0,5)
Betaproteobacteria	14	(4,38,20,2)	10	(35,6,7,32)
Gammaproteobacteria	2	(0,2,2,0)	4	(9,0,4,9)
Deltaproteobacteria	25	(6,43,55,10)	12	(47,11,9,48)
Cyanobacteria	**8**	**(3,27,38,7))**	**1**	**(1,0,0,1)**
Bacteroidetes	**7**	**(0,13,11,0)**	**4**	**(4,0,0,4)**
Actinobacteria	**7**	**(0,9,9,1)**	**12**	**(23,6,4,28)**
Acidobacteria	**5**	**(0,6,6,0)**	**7**	**(10,0,0,7)**
Chloroflexi	**5**	**(0,10,8,0)**	**3**	**(4,0,0,6)**
Verrucomicrobia	**2**	**(0,2,2,0)**	**2**	**(2,0,0,3)**
Spirochaetes	**1**	**(0,1,2,0)**	**0**	**-**
Firmicutes	**1**	**(0,2,1,0)**	**1**	**(1,0,0,1)**
Gemmatimonadetes	**1**	**(0,1,1,0)**	**2**	**(9,1,2,11)**
Planctomycetes	**0**	**-**	**3**	**(4,0,0,3)**
Nitrospira	**0**	**-**	**1**	**(3,2,2,4)**
**Unclassified Bacteria**	**11**	**(0,18,17,0)**	**12**	**(23,1,4,25)**
**Total[b]**	**95**	**(13,171,188,20)**	**78**	**(180,27,32,187)**

[a] A Number represents the total number of shared OTUs in each phylogenetic group, and the numbers in parenthesis are the numbers of clones in the each OTUs originated from the four libraries (BD1R, BW1R, BW2R, and BD2R).

[b] Total numbers of shared OTUs and sequences at phylum level.

[c] OTUs specific to the libraries BW1R and BW2R.

[d] OTUs specific to the libraries BD1R and BD2R.

Table S3. Taxonomic distribution of bacterial sequences at family-level resolution.

Phylum	Family	Ratio per total sequences (%) †				
		BD1R	BW1R	BW2R	BD2R	BW1D
Proteobacteria	*Geobacteraceae*	9.45	5.86	5.29	5.13	5.61
Chloroflexi	*Anaerolineaceae*	5.52	5.67	5.19	3.94	15.82
Proteobacteria	*Cystobacteraceae*	3.65	4.16	4.72	3.02	2.00
Proteobacteria	Burkholderiales_incertae_sedis	2.43	3.31	5.29	3.48	0.20
Cyanobacteria	Family I	1.03	2.93	3.12	2.29	0.00
Proteobacteria	*Rhodocyclaceae*	1.59	2.08	1.23	1.37	1.30
Acidobacteria	*Holophagaceae*	0.84	1.42	0.85	0.64	0.70
Proteobacteria	*Sinobacteraceae*	0.84	1.42	1.32	0.37	0.90
Gemmatimonadetes	*Gemmatimonadaceae*	2.25	1.42	2.46	2.75	2.10
Planctomycetes	*Planctomycetaceae*	2.62	1.23	0.66	2.01	1.40
Actinobacteria	*Acidimicrobiaceae*	0.28	1.13	0.76	1.01	0.20
Proteobacteria	*Pseudomonadaceae*	1.22	1.13	1.04	0.55	0.10
Proteobacteria	*Syntrophobacteraceae*	0.19	0.95	0.57	0.09	0.50
Proteobacteria	*Acetobacteraceae*	0.37	0.95	1.61	0.46	0.10
Proteobacteria	*Polyangiaceae*	1.96	0.85	0.47	3.11	0.50
Proteobacteria	*Comamonadaceae*	0.37	0.85	1.79	0.82	0.20
Bacteroidetes	*Chitinophagaceae*	1.03	0.85	0.19	0.27	2.20
Proteobacteria	*Syntrophaceae*	0.09	0.66	0.85	0.82	0.70
Actinobacteria	*Kineosporiaceae*	0.28	0.57	0.00	0.92	0.00
Proteobacteria	*Syntrophorhabdaceae*	0.00	0.57	0.57	0.09	0.20
Firmicutes	*Bacillaceae 1*	0.00	0.57	0.09	0.09	0.50
Armatimonadetes	*Chthonomonadaceae*	0.75	0.57	1.04	1.01	1.10
Proteobacteria	*Rhodospirillaceae*	0.65	0.47	0.57	0.73	0.10
Proteobacteria	*Alcaligenaceae*	0.75	0.47	1.04	1.56	0.10
Proteobacteria	*Desulfobulbaceae*	0.19	0.38	0.76	0.09	0.40
Proteobacteria	*Methylocystaceae*	0.37	0.38	0.19	0.09	0.00
Proteobacteria	*Oxalobacteraceae*	0.09	0.38	0.28	0.27	0.20
Bacteroidetes	*Cryomorphaceae*	0.09	0.38	0.28	0.00	0.10
Actinobacteria	*Intrasporangiaceae*	0.28	0.28	0.00	0.09	0.00
Actinobacteria	*Nocardioidaceae*	0.47	0.28	0.47	0.37	0.00
Actinobacteria	*Pseudonocardiaceae*	0.00	0.28	0.09	0.09	0.00
Proteobacteria	*Hyphomicrobiaceae*	0.19	0.28	0.09	0.46	0.00
Proteobacteria	*Xanthomonadaceae*	0.47	0.28	0.66	0.46	1.20
Nitrospira	*Nitrospiraceae*	0.56	0.28	0.28	0.64	0.30
Chlorobi	*Ignavibacteriaceae*	0.47	0.28	0.19	0.00	0.30
Actinobacteria	Acidimicrobineae_incertae_sedis	0.28	0.19	0.28	0.18	0.40
Actinobacteria	*Micromonosporaceae*	0.19	0.19	0.00	0.92	0.00
Actinobacteria	*Thermomonosporaceae*	0.00	0.19	0.09	0.00	0.00
Proteobacteria	*Bdellovibrionaceae*	0.09	0.19	0.09	0.27	0.10
Proteobacteria	*Bradyrhizobiaceae*	0.28	0.19	0.09	0.27	0.30
Proteobacteria	*Caulobacteraceae*	0.19	0.19	0.09	0.18	0.00
Proteobacteria	*Neisseriaceae*	0.00	0.19	0.09	0.09	0.00
Firmicutes	*Clostridiaceae 1*	0.00	0.19	0.28	0.18	0.10
Spirochaetes	*Spirochaetaceae*	0.00	0.19	0.76	0.18	0.60
Spirochaetes	*Leptospiraceae*	0.00	0.19	0.00	0.00	0.00
Actinobacteria	*Conexibacteraceae*	0.00	0.09	0.09	0.18	0.00
Actinobacteria	*Geodermatophilaceae*	0.65	0.09	0.19	0.46	0.00
Actinobacteria	*Microbacteriaceae*	0.00	0.09	0.00	0.00	0.00
Actinobacteria	*Mycobacteriaceae*	0.00	0.09	0.00	0.18	0.00
Proteobacteria	*Desulfobacteraceae*	0.19	0.09	0.28	0.09	0.00
Proteobacteria	*Phaselicystidaceae*	0.00	0.09	0.19	0.09	0.00
Proteobacteria	*Kofleriaceae*	0.09	0.09	0.19	0.18	0.00
Proteobacteria	*Desulfovibrionaceae*	0.00	0.09	0.00	0.00	0.10

Continued on the following page.

Proteobacteria	*Rhodobiaceae*	0.28	0.09	0.19	0.09	0.00
Proteobacteria	*Xanthobacteraceae*	0.00	0.09	0.00	0.18	0.10
Proteobacteria	*Rhodobacteraceae*	0.00	0.09	0.28	0.00	0.00
Proteobacteria	*Hydrogenophilaceae*	0.09	0.09	0.09	0.00	0.00
Proteobacteria	*Methylococcaceae*	0.09	0.09	0.38	0.00	0.20
Firmicutes	*Gracilibacteraceae*	0.00	0.09	0.00	0.00	0.00
Firmicutes	*Pasteuriaceae*	0.00	0.09	0.19	0.55	0.10
Firmicutes	*Veillonellaceae*	0.00	0.09	0.00	0.00	0.10
Chloroflexi	*Chloroflexaceae*	0.09	0.09	0.19	0.18	0.00
Armatimonadetes	*Armatimonadaceae*	0.09	0.09	0.00	0.00	0.00
Verrucomicrobia	*Opitutaceae*	0.00	0.09	0.38	0.18	0.10
Cyanobacteria	Family XI	0.09	0.09	0.76	0.00	0.00
Cyanobacteria	Family II	0.00	0.09	0.19	0.00	0.00
Bacteroidetes	*Sphingobacteriaceae*	0.00	0.09	0.09	0.00	0.10
Bacteroidetes	*Cyclobacteriaceae*	0.00	0.09	0.28	0.00	0.10
Spirochaetes	*Brevinemataceae*	0.00	0.09	0.09	0.00	0.00
Deinococcus-Thermus	*Deinococcaceae*	0.00	0.09	0.00	0.00	0.00
Actinobacteria	*Iamiaceae*	0.37	0.00	0.00	0.09	0.00
Actinobacteria	*Nakamurellaceae*	0.19	0.00	0.09	0.18	0.00
Actinobacteria	*Cryptosporangiaceae*	0.00	0.00	0.09	0.00	0.00
Actinobacteria	*Sporichthyaceae*	0.00	0.00	0.09	0.18	0.00
Actinobacteria	*Micrococcaceae*	0.00	0.00	0.09	0.09	0.20
Actinobacteria	*Propionibacteriaceae*	0.00	0.00	0.00	0.27	0.00
Actinobacteria	*Streptomycetaceae*	0.19	0.00	0.38	0.64	0.10
Actinobacteria	*Coriobacteriaceae*	0.00	0.00	0.00	0.27	0.30
Proteobacteria	*Desulfuromonadaceae*	0.09	0.00	0.09	0.00	0.20
Proteobacteria	*Nannocystaceae*	0.00	0.00	0.09	0.09	0.00
Proteobacteria	*Bacteriovoracaceae*	0.09	0.00	0.28	0.00	0.00
Proteobacteria	Rhizobiales_incertae_sedis	0.00	0.00	0.09	0.00	0.00
Proteobacteria	*Rhizobiaceae*	0.00	0.00	0.09	0.00	0.10
Proteobacteria	*Beijerinckiaceae*	0.00	0.00	0.00	0.09	0.00
Proteobacteria	*Sphingomonadaceae*	0.00	0.00	0.09	0.09	0.30
Proteobacteria	*Burkholderiaceae*	0.19	0.00	0.09	0.18	0.10
Proteobacteria	*Methylophilaceae*	0.00	0.00	0.09	0.00	0.00
Proteobacteria	*Moraxellaceae*	0.00	0.00	0.00	0.00	0.10
Proteobacteria	*Coxiellaceae*	0.00	0.00	0.09	0.00	0.10
Proteobacteria	*Aeromonadaceae*	0.00	0.00	0.09	0.00	0.00
Proteobacteria	*Ectothiorhodospiraceae*	0.00	0.00	0.00	0.00	0.20
Proteobacteria	*Thiotrichales_incertae_sedis*	0.00	0.00	0.00	0.00	0.10
Firmicutes	*Ruminococcaceae*	0.00	0.00	0.47	0.00	0.40
Firmicutes	Incertae Sedis III	0.00	0.00	0.00	0.09	0.00
Firmicutes	Clostridiales_Incertae Sedis XVIII	0.00	0.00	0.00	0.00	0.10
Firmicutes	*Paenibacillaceae 1*	0.00	0.00	0.09	0.09	0.00
Firmicutes	*Thermoactinomycetaceae 1*	0.00	0.00	0.00	0.09	0.00
Elusimicrobia	*Elusimicrobiaceae*	0.09	0.00	0.19	0.00	0.20
Chloroflexi	*Caldilineaceae*	0.00	0.00	0.00	0.00	0.10
Planctomycetes	*Phycisphaeraceae*	0.19	0.00	0.00	0.09	0.10
Verrucomicrobia	*Verrucomicrobiaceae*	0.09	0.00	0.09	0.00	0.10
Cyanobacteria	Family IV	0.00	0.00	0.47	0.00	0.00
Cyanobacteria	Family XIII	0.00	0.00	0.09	0.00	0.00
Cyanobacteria	Family IX	0.00	0.00	0.00	0.09	0.00
Cyanobacteria	Family VIII	0.00	0.00	0.00	0.09	0.00
Bacteroidetes	*Cytophagaceae*	0.00	0.00	0.00	0.09	0.00
Bacteroidetes	*Porphyromonadaceae*	0.00	0.00	0.19	0.00	0.00
Bacteroidetes	*Flavobacteriaceae*	0.00	0.00	0.09	0.00	0.20
Lentisphaerae	*Victivallaceae*	0.00	0.00	0.09	0.00	0.00
	unclassified family	54.44	52.17	46.46	53.39	55.56

† listed in order of ratio in BD1R.

Table S4. Taxonomic distribution of archaeal sequences at family-level resolution.

		Ratio per total sequences (%) †				
**Phylum**	**Family**	**AD1R**	**AW1R**	**AW2R**	**AD2R**	**AW1D**
Euryarchaeota	*Methanosaetaceae*	33.68	32.40	32.70	41.03	16.62
Euryarchaeota	*Methanosarcinaceae*	18.45	11.11	9.06	20.02	7.48
Euryarchaeota	Methanomicrobiales_incertae_sedis	3.31	6.35	7.88	3.99	9.60
Euryarchaeota	*Methanospirillaceae*	0.00	0.37	0.27	0.00	0.18
Euryarchaeota	*Methanomicrobiaceae*	0.00	0.28	0.18	0.09	0.18
Euryarchaeota	*Methanocellaceae*	3.69	9.90	8.88	6.25	16.07
Euryarchaeota	*Methanobacteriaceae*	1.23	0.84	1.00	0.54	1.48
Euryarchaeota	Thermoplasmatales_incertae_sedis	0.57	0.56	0.82	0.18	1.20
Crenarchaeota	*Fervidicoccaceae*	0.47	0.19	0.45	0.27	0.00
	unclassified family	38.60	38.00	38.77	27.63	47.18

† listed in order of ratio in AD1R.

Fig. S1

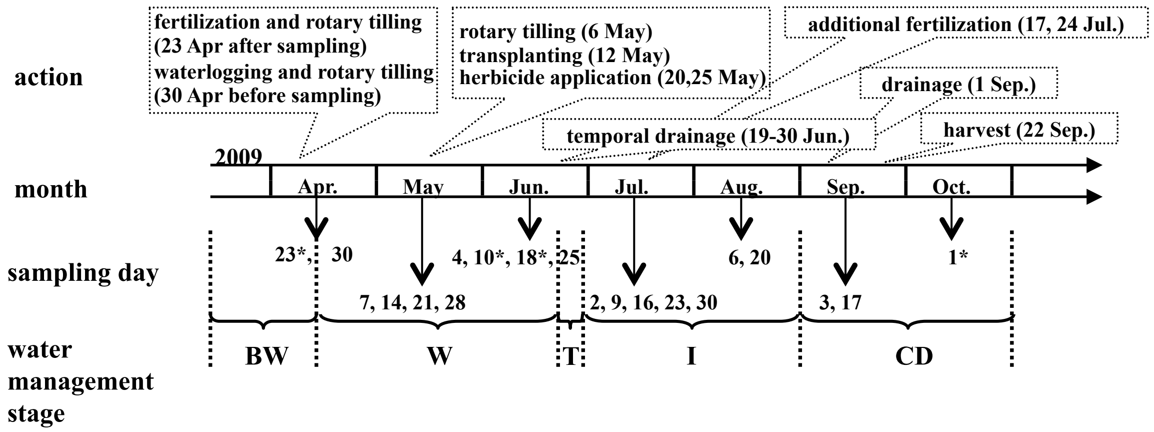

Fig. S1. Water management, rice cultivation, and sampling histories in the paddy field used in this study. Water management periods were separated to five stages: before waterlogging (stage BW; until 29 April), waterlogging (stage W; April 30–June 18), temporal drainage (stage T; June 19–30), intermittent drainage (stage I; July 1–August 31), and after complete drainage (stage CD; after September 1). Asterisks (*) indicate the samples used for the clone library analysis.

Fig. S2

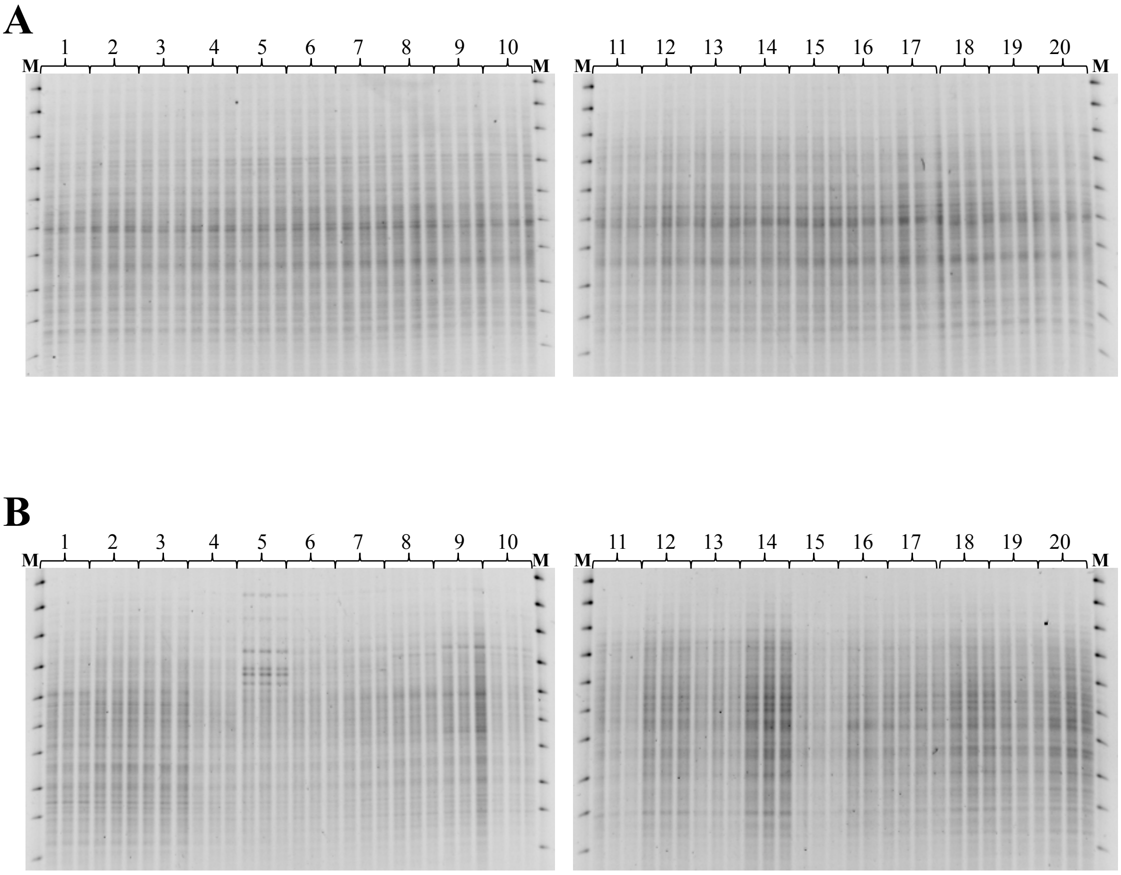

Fig. S2. Reproducibility of DGGE profiles of bacterial communities based on DNA (A) or RNA samples (B) extracted with three replications. Numbers of the each lane at the DGGE profiles represent soil samples, 1, 0423; 2, 0430; 3, 0507; 4, 0514; 5, 0521; 6, 0528; 7, 0604, 8, 0610; 9, 0618; 10, 0625; 11, 0702; 12, 0709; 13, 0716; 14, 0723; 15, 0730; 16, 0806; 17, 0820; 18, 0903; 19, 0917; 20, 1001. These numbers in each sample ID represent the sampling date (month + day) described in the Fig. S1, eg;. 0423 and 0723 indicate the soil sample collected on 23 April and 23 July respectively. Lane M shows the profiles of DGGE Maker II (Nippon Gene, Tokyo, Japan).

Fig. S3

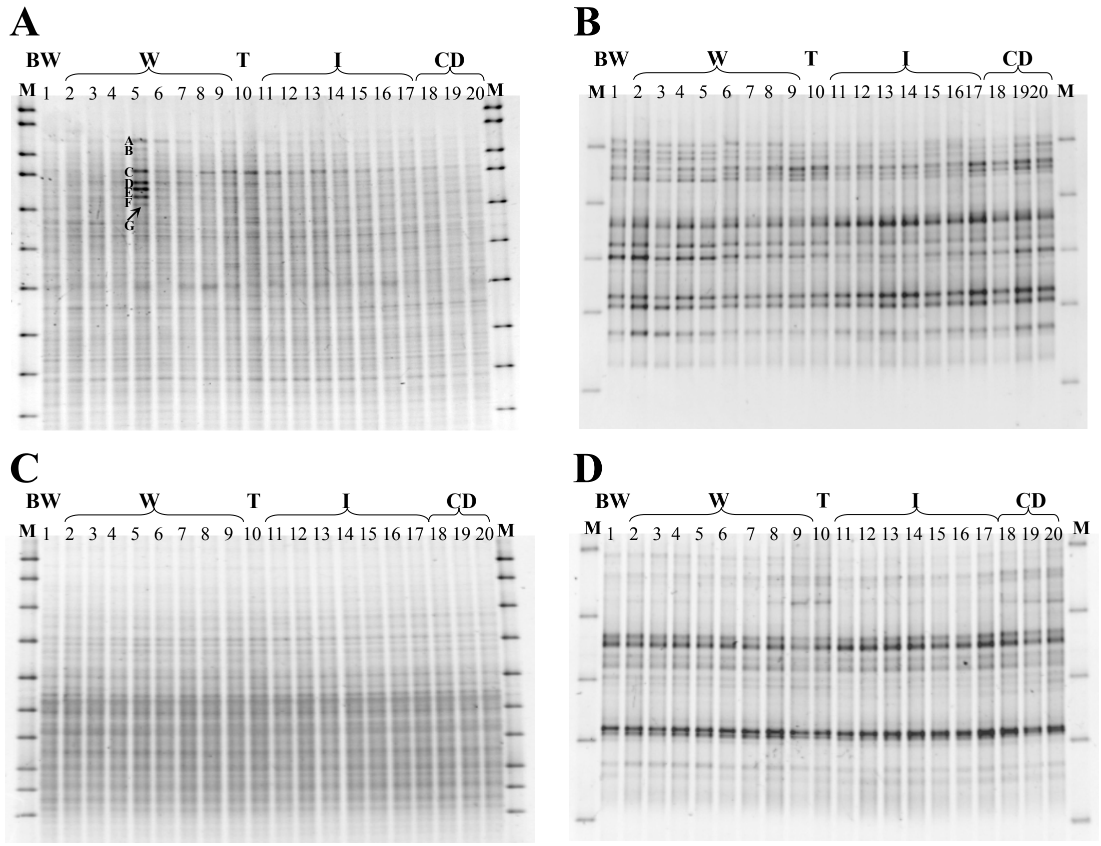

Fig. S3. DGGE profiles of bacterial and archaeal communities. DGGE profiles of A) RNA-based DGGE targeting bacterial 16S rRNA; B) RNA-based DGGE targeting archaeal 16S rRNA; C) DNA-based DGGE targeting bacterial 16S rRNA gene; and D) DNA-based DGGE targeting archaeal 16S rRNA gene are shown. Numbers of the each lane at the DGGE profiles represent soil samples as described in legend of Fig. S2. Lane M shows the profiles of DGGE Maker II (Nippon Gene). DGGE bands indicated by alphabets (A to G) were excised from the gel for sequence analysis.

Fig. S4

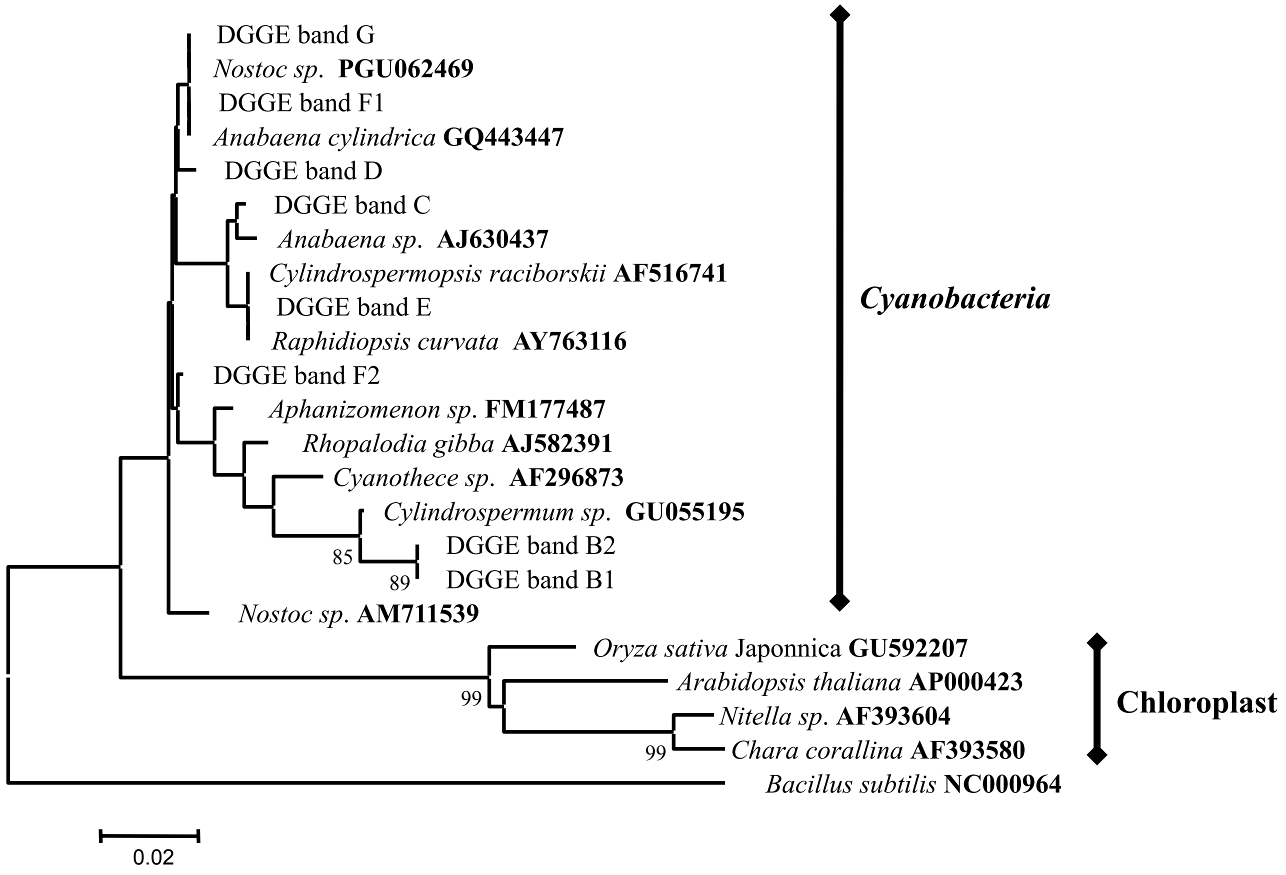

Fig. S4. Phylogenetic tree constructed based on the sequences obtained from the DGGE bands (Fig. S3A) and reference cyanobacterial sequences. Bootstrap values (>70%) with 1000 replicates are shown next to the branches. The 16S rRNA sequence of Bacillus subtilis (NC_00964) was used as an outgroup.
